# Sensor feedback system enables automated deficit irrigation scheduling for cotton

**DOI:** 10.3389/fpls.2023.1149424

**Published:** 2023-03-09

**Authors:** Susan A. O’Shaughnessy, Paul D. Colaizzi, Craig W. Bednarz

**Affiliations:** ^1^ Conservation and Production Research Laboratory, USDA-ARS, Bushland, TX, United States; ^2^ Semi-arid Agricultural Systems Institute and West Texas A&M University, Canyon, Amarillo, TX, United States; ^3^ Texas A&M AgriLife Research, Amarillo, TX, United States

**Keywords:** crop water stress index (CWSI), infrared thermometry, soil water sensing, wireless sensor networks, variable rate irrigation (VRI)

## Abstract

Precision irrigation technologies using sensor feedback can provide dynamic decision support to help farmers implement DI strategies. However, few studies have reported on the use of these systems for DI management. This two-year study was conducted in Bushland, Texas to investigate the performance of the geographic information (GIS) based irrigation scheduling supervisory control and data acquisition (ISSCADA) system as a tool to manage deficit irrigation scheduling for cotton (*Gossypim hirsutum* L). Two different irrigation scheduling methods automated by the ISSCADA system — (1) a plant feedback (designated C) - based on integrated crop water stress index (_i_CWSI) thresholds, and (2) a hybrid (designated H) method, created to combine soil water depletion and the _i_CWSI thresholds, were compared with a benchmark manual irrigation scheduling (M) that used weekly neutron probe readings. Each method applied irrigation at levels designed to be equivalent to 25%, 50% and 75% replenishment of soil water depletion to near field capacity (designated I_25_, I_50_ and I_75_) using the pre-established thresholds stored in the ISSCADA system or the designated percent replenishment of soil water depletion to field capacity in the M method. Fully irrigated and extremely deficit irrigated plots were also established. Relative to the fully irrigated plots, deficit irrigated plots at the I_75_ level for all irrigation scheduling methods-maintained seed cotton yield, while saving water. In 2021, the irrigation savings was a minimum of 20%, while in 2022, the minimum savings was 16%. Comparing the performance of deficit irrigation scheduling between the ISSCADA system and the manual method showed that crop response for all three methods were statistically similar at each irrigation level. Because the M method requires labor intensive and expensive use of the highly regulated neutron probe, the automated decision support provided by the ISSCADA system could simplify deficit irrigation management of cotton in a semi-arid region.

## 1 Introduction

More than 508,000 ha were dedicated to cotton production in the Texas North High Plains region in 2019 (Hundl, 2020), where agricultural production accounts for greater than 90% of total water use. Water for this region is mainly sourced from the Ogallala Aquifer, however, saturated thickness in this area is highly depleted, leading to water allocation restrictions and fields with limited well capacity (Scanlon et al., 2012; Mitchell-McCallister et al., 2021). Due to the dwindling supply of water, maximizing its value in this region is critical, but not easy to achieve due to high evaporative demand, and climate variability. Since 1995, acreage planted to upland cotton has increased by 21%, while area planted to corn (maize) has remained stable in Texas (Hundl, 2020). Cotton can be an appropriate choice of crop to maximize water productivity as it requires less water than maize and can be grown at evapotranspiration levels less than maximum potential (Fereres and Soriano, 2007). It’s cultivation on farms with depleted well capacities could be a profitable crop for farmers in the Texas High Plains region (Fan et al., 2022). However, there are inherent risks with cotton production in this area and when using DI management on any crop. The three main risks are: 1) limited heat units in the spring and autumn seasons, 2) unpredictable precipitation events - whereby excessive rainfall late in the season can promote extensive vegetative growth and slow boll maturation, while intense rainfall and hail early in the season can decimate the crop, and 3) high evaporative demand during the summer months and extended periods of drought, which can significantly reduce lint yields (Tolk and Howell, 2010; Bordovsky, 2020; Lu et al., 2020). When practicing DI, inadequate irrigation during the vegetative stage of some cotton cultivars can reduce plant height, the number of nodes and the dry weights of leaves and stems (Pace et al., 1999; Papastylianou and Argyrokastritis, 2014). Deficit irrigation can also negatively impact seed cotton yields during the reproductive stages by reducing flowering and boll retention (Grimes et al., 1969; Guinn and Mauney, 1984a; Guinn and Mauney, 1984b). Therefore, farmers practicing DI strategies must closely assess evaporative demand and crop growth stage, while making timely irrigation scheduling decisions to prevent significant yield loss.

Precision irrigation technologies that use sensor feedback can provide automated decision support for irrigation scheduling (DSSIS) that could be used to help farmers implement DI strategies for cotton. Chen et al. (2020) developed a DSSIS that used forecasted rainfall and a water stress index simulated by the Root Zone Quality Model to schedule irrigations. The system improved lint yield and water productivity as compared with a soil moisture sensor-based irrigation scheduling method. Vellidis et al. (2008) developed a wireless soil water sensing system to provide real-time site-specific irrigation scheduling for cotton. Another sensor-based irrigation scheduling system is the Irrigation Scheduling Supervisory Control and Data Acquisition (ISSCADA) System patented by Evett et al. (2014). The early ISSCADA system triggered an irrigation for plant feedback-controlled management zones (MZs) when the unique thermal stress threshold for each of the three designated irrigation treatments, 75%, 50% and 25% of full, was exceeded. The irrigation depth applied was the same for each treatment level. Results showed that the ISSCADA system produced lint yields that were similar or better than lint yields from manually irrigated treatment plots, and irrigation water use efficiency was similar between irrigation methods when compared at the same irrigation level (O’Shaughnessy et al., 2015). In a recent study, Vories et al. (2021) used a mature ISSCADA system embodied in ARSPivot for irrigation scheduling of cotton in a sub-humid climate, and compared the plant feedback method with the Arkansas Irrigation Scheduler (AIS). They reported that irrigation water productivity (economic yield/unit of irrigation applied) was significantly greater for cotton managed by the ISSCADA system as compared with the AIS method.

In the Texas High Plains region, most irrigated acres are managed with sprinkler irrigation systems drawing from the Ogallala Aquifer (Colaizzi et al., 2009b). Providing deficit irrigation scheduling strategies for cotton producers in this region is critical to help sustain the regional economy (Crouch et al., 2020). Although an early plant feedback method was used to schedule irrigations for cotton in Bushland, Texas, the updated ISSCADA system using plant feedback and the combination of plant feedback and soil water sensing have not been tested on cotton in this region. The objectives of this study were to: 1) compare cumulative irrigation and crop response between deficit irrigated treatments, using the ISSCADA and manual irrigation scheduling methods, with fully irrigated treatment plots, and 2) compare cumulative irrigation amounts and crop response of deficit irrigated cotton between the manual and the ISSCADA-plant feedback and ISSCADA-hybrid irrigation scheduling methods. Performances were evaluated by comparing seed cotton yield, seasonal crop water use (ET_c_), crop water productivity (CWP) and irrigation water use efficiency (IWUE).

## 2 Materials and methods

### 2.1 Location and experimental design

**Figure 1:**
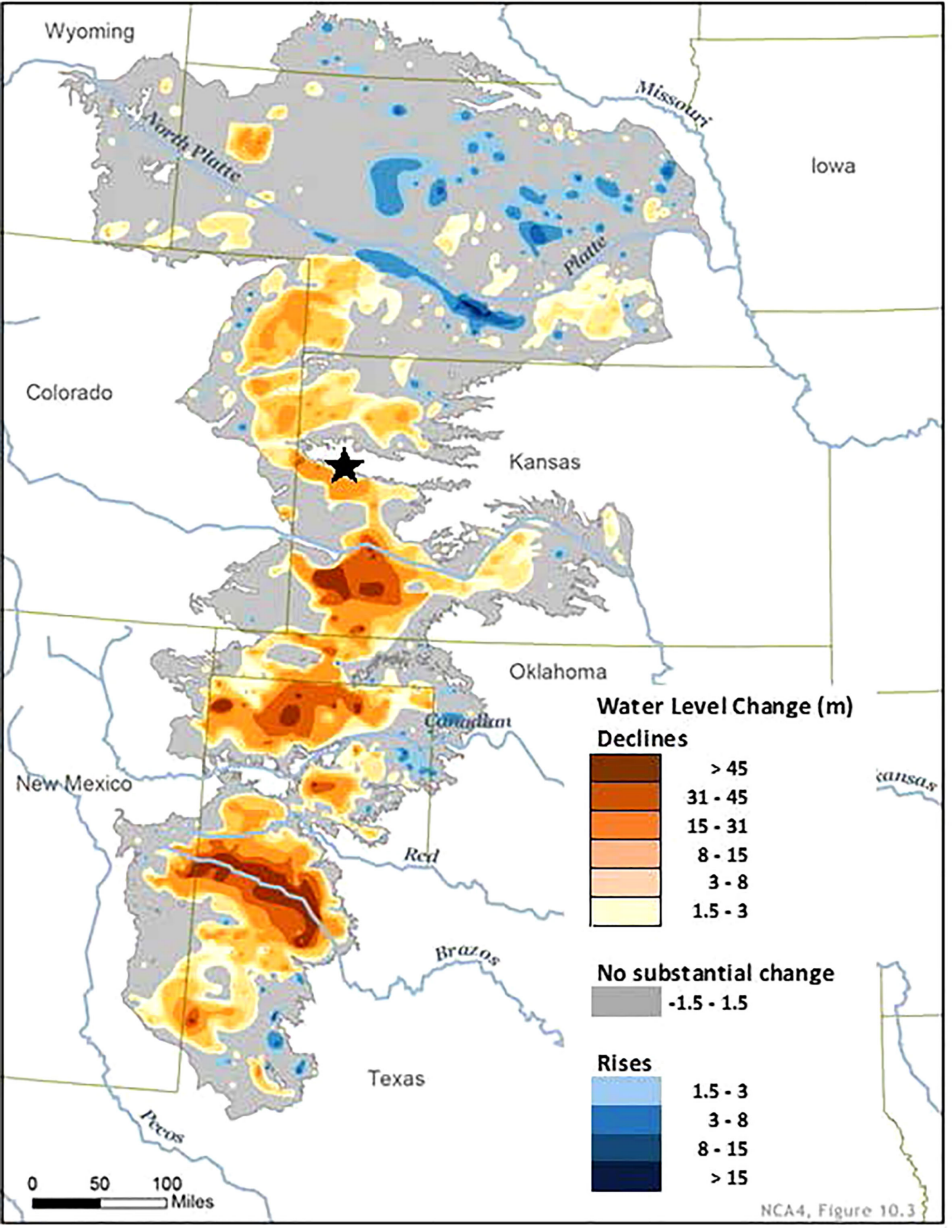
Location of the study (designated by the star) against a map showing the declining levels of the Ogallala Aquifer from the period before the aquifer was drawn down (circa 1950) to 2015 (NOAA, 2019).

The study was conducted at the Conservation and Production Research Laboratory (CPRL) in Bushland, Texas (35° 10’2.42” N, 102°5’32.5” W, elevation 1142 m). The site is in a semi-arid region with mean annual rainfall of approximately 400 mm, and variable rainfall during the cropping season (May – September). The soils were Pullman clay loam fine, superactive, mixed, thermic torretic Paleustoll, (Unger and Pringle, 1981). The soil is slowly permeable with a hard pan layer of calcium carbonate at approximately 1.5 m and the plant-available water holding capacity to this depth is roughly 210 mm (Evett et al., 2019). Mean bulk density ranges from approximately 1. 4 to 1.7 Mg m^-3^ (Evett et al., 2022). The source of irrigation water is the Ogallala Aquifer. Practicing deficit irrigation in the surrounding region is important as saturated thickness of the aquifer in this region is depleted between 7 to 15 m (**Figure 1**).

Cotton (*Gossypium hirsutum* L.), variety Phytogen 210 W3FE, was planted on June 4, 2021, day of year (DOY 155) and on May 13, 2022, (DOY 133) under one-half of a center pivot field (10.5 ha) and irrigated with a six-span center pivot sprinkler system. The cotton variety was an early maturing genotype. Critical growth stages for the two years were offset by the difference in planting dates (**Table 1**).

**Table 1:** Agronomic data and observed critical growth stages for the 2021 and 2022 growing seasons.

	2021	2022
Fertilizer	168 kg N ha^-1^, 67 kg P ha^-1^	129 kg N ha^-1^, 79 kg P ha^-1^
Planting Date	June 4 (DOY 155)	May 13 (DOY 133)
Planting Rate	21 seeds m^-2^	21 seeds m^-2^
Herbicides	June 7: S-metolachlor, 0.86 l ha^-1^ July 21: Glyphosate 1.5 l ha^-1^	May 16: S-metolachlor, 0.86 l ha^-1^ June 28: Glyphosate 1.5 l ha^-1^
Pesticides	June 19: Chlorpyrifos (Govern 4E) 1.2 l ha^-1^	June 10: Chlorpyrifos 1.2 l ha^-1^
Square formation	Jul 30	Jul 10
White bloom	Aug 9	Jul 23
Boll formation	Aug 31	Aug 11
First open boll	Sep 29	Sep 6
Harvest Dates	Nov 1 – Nov 5	Oct 1 – Oct 18

### 2.2 Irrigation sprinkler and sensor hardware

The center pivot irrigation system was a six-span Valley 8000 series with a pivot lateral length of 286 m. The sprinkler was equipped with a commercial zone control variable rate irrigation (VRI) hardware package, a GPS unit at the end tower, and the Irrigation Supervisory Control and Data Acquisition (ISSCADA) system, patented by ARS scientists (Evett et al., 2014) and described in detail in Andrade et al. (2020). The irrigation scheduling methods used by the ISSCADA system are summarized under “Irrigation Scheduling Methods” later in this section.

For this study, two sprinkler zones were combined to operate as one control zone. Each control zone contained 12 drop hoses and measured a total of 18.3 m wide. Irrigation was applied using low elevation spray application (LESA) with low-drift nozzles and multi-trajectory spray plate assemblies (3030 Series, 3NV, Nelson, Walla Walla, WA) elevated approximately 0.46 m above the ground. Irrigation buffers in the shape of pie-slices were established at the beginning and end of each half of the cropped field to ensure the VRI system had time to synchronize the operation of its zone-controlled sprinkler banks at the start of an irrigation and to provide a cropped buffer area for the treatment plots.

An embedded rugged computer (model MXE-1401, ADLINK Technology, San Jose, CA) with a cellular modem was located at the pivot point and connected to the control panel of the center pivot sprinkler with an RS-232 cable. Also connected to the computer were two radio modems- the SAPIP coordinator (Dynamax, Inc., Houston, TX), and a spread spectrum radio (model RF-407, 900 MHz, Campbell Scientific, Logan, UT). A total of 10 infrared thermometers (IRTs) (model- SAPIP-IRT, Dynamax, Inc., Houston, TX) were mounted on the pivot lateral. Two pairs of IRTs were mounted on brackets near the borders of each control zone and pointed towards the center of the zone. In addition, one IRT was located in each of the M_100_ treatment plots. These data were averaged and used as the reference temperature curve to scale data from the moving network of IRTs on the pivot lateral. The IRTs measured object temperature, sensor body temperature, and battery voltage every 6 s. One-minute averaged data packets were transmitted to the computer at the pivot point. More details on the IRTs and the IRT network communication protocol are found in O’Shaughnessy et al. (2011).

A soil water sensing station was located in each type of ISSCDA-hybrid treatment plot (H_75_, H_50_ and H_25_) to provide input of soil water depletion levels. The soil water sensing stations were established as a distributed wireless sensor network, where each station contained a datalogger (CR300 with an internal RF-407 radio, Campbell Scientific, Logan, UT) and transmitted data hourly to the base station RF-407 radio connected to the embedded computer at the pivot point. Each datalogger was powered by a sealed lead acid battery that was recharged with a 10-W solar panel. A trench was dug in a furrow and four soil water sensors, time domain reflectometers (TDRs), (model TDR-315L or 315H, Acclima, Meridian, ID) were installed horizontally near the cotton plants at depths of 10 cm, 20 cm, 30 cm, and 50 cm. Data from the TDRs were acquired every 1-minute, averaged every 15-minutes. Each soil water sensing station was within 1 m of the neutron access tube installed in the treatment plot.

A standalone weather station was located nearby the center pivot field. Air temperature, relative humidity, solar irradiation, wind speed and wind direction were collected with the base-station datalogger (CR1000X, Campbell Scientific, Logan, UT). All micrometeorological data were measured every 5 s, averaged every 1-minute and transmitted hourly to the computer at the pivot point. The embedded computer was accessed in the office through the cellular connection using the ‘Remote Desktop’ application. Accessing the embedded computer allowed for monitoring data collection, reviewing and uploading prescription maps and starting an irrigation or scan (running the sprinkler dry across the field). All center pivot operations were conducted with the ARSPivot software, which resided on the embedded computer.

### 2.3 Agronomics

Fertilizer was applied using a knife rig to meet a yield goal of 682 kg ha^-1^ of cotton based on test results from composite soil samples taken in March of 2021 and 2022 from the W-SW and N-NE halves of the field, respectively (**Table 1**). Test results were from a commercial soil testing laboratory. Pre-plant irrigations were applied to help enable germination. Two pre-plant irrigations were applied in 2021 in late April, and four pre-plant irrigations were applied in 2022 due to a dry winter and spring season. Each irrigation event was 25 mm. In both years, cotton was planted at a rate of 21 seeds m^-2^ and 2.5 cm deep in circular rows spaced 0.76 m apart using a GPS guided planter. The W-SW half of the center pivot field was cropped in 2021, while in 2022, the N-NE half of the field was cropped; in both years, planting was on ground fallowed in the previous season. Post-plant herbicides were applied to control weeds, mainly pigweed (Amaranthus spp.) and Devil’s Claw (Harpagophytum spp.). The pesticide, Govern 4E, was applied to avert extensive damage from thrips (Thysanoptera) (**Table 1**).

**Figure 2:**
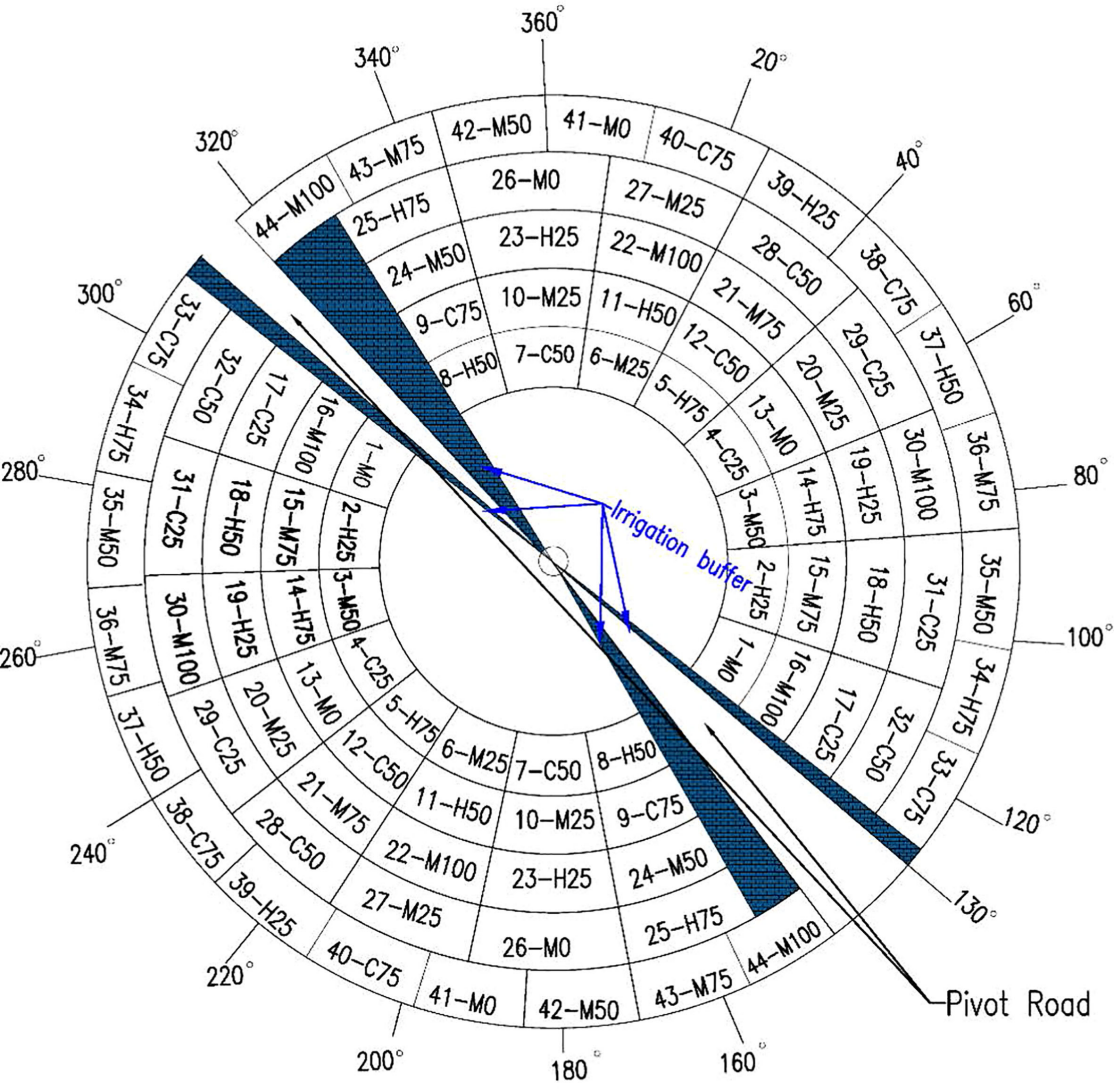
Plot plan for the two-year cotton study- the W-SW half of field was cropped from 310° to 140° in 2021, and the N-NE half of field was cropped from 130°-316° for the 2022 growing season.

For each growing season, the field was divided into 44 treatment plots (MZs), where each treatment- M_100_, M_75_, M_50_, M_25_, M_0_, H_75_, H_50_, H_25_, C_75_, C_50_ and C_25_ was replicated four times and arranged in a complete randomized design (**Figure 2**). The labels “M”, “H”, and “C” designate the irrigation scheduling methods- manual (M), the ISSCADA-hybrid (H), and the ISSCADA-plant feedback I system, respectively. The numeric values designate the irrigation treatment level, I_100_, I_75_, I_50_, and I_25_. The length of each plot varied depending on the radial distance of the control zone from the pivot point. The width of each plot was 18.2 m and plot lengths varied from 32.0 m to 57.3 m at half width, depending on their distance from the pivot point. A neutron access tube was installed near the center of each plot to a depth of 3.0 m. Plant height and width measurements were recorded starting in the second week of July and continued biweekly through the last week in August. All agronomic measurements and hand-harvested samples were taken in each treatment plot from a 10 m^2^ area (4 rows x 328 cm) centered around the access tube.

### 2.4 Irrigation scheduling methods

#### 2.4.1 Manual

For the treatment plots designated under manual control, irrigation was applied to replenish a percentage of soil water depletion to near field capacity (96% of full in the top 1.5 m of the soil profile). The irrigation amount was determined by averaging weekly neutron probe measurements in the fully irrigated treatment plots, designated M_100_. Irrigation was replenished at 25%, 50%, 75% and 100% of soil water depletion to field capacity in the top 150 cm of the soil profile for treatment plots designated M_25_, M_50_, M_75_ and M_100_, respectively. The effective rooting depth of cotton was assumed to be 150 cm due to the existing hard pan layer at this depth. Treatment plots where irrigation was withheld after the plant stand was established were designated M_0_. The neutron probe (NP) (model 503DR, InstroTek Inc., formerly Campbell Pacific Nuclear, Concord, Calif., USA) readings were taken weekly in the M_100_ treatment plots and averaged to determine the amount to irrigate the treatment plots under manual control. The NP was field calibrated in 2021 to an accuracy better than 0.01 m^3^ m^-3^, resulting in separate calibrations for the three distinct soil horizons (Ap, Bt, and Btk) (Evett et al., 2022). Readings were taken from 10 to 230 cm depths in 20-cm increments using the method described in Evett (2008). Neutron probe measurements were also used to calculate seasonal crop water use (ET_c_) for all treatment plots based on the soil water balance. Therefore, NP measurements were taken when access tubes were installed (July1, 2021 and July 13, 2022), at harvest for all treatment plots, and every 30 days in those treatment plots receiving less than full irrigation.

#### 2.4.2 ISSCADA scheduling methods

##### 2.4.2.1 Plant feedback

This irrigation scheduling method uses data from the reference temperature curve discussed in Peters and Evett (2004) to scale canopy temperature data from one-time-of-day measurements made from the moving network of IRTs on the center pivot lateral. Scaling the one-time-of-day temperature measurements provided one-minute estimates of diurnal canopy temperature data. This one-minute data (from 0900 hrs to 1900 hrs) was matched by time stamp with the collected micrometeorological data from the nearby weather station to calculate a theoretical crop water stress index (CWSI), where the meteorological data were used to estimate the upper and lower limits of the stress index (Jackson et al., 1981). The integration of the daily one-minute CWSI values is characterized in equation 1:


(1) $$iCWSI=\;\int_{09:00}^{19:00}\left[\frac{\left(T_{s}-\;T_{a}\right)-\left(T_{ll}\right)}{\left(T_{ul}\right)-\left(T_{ll}\right)}\right]\;dt$$

where *T_s_* [°C] was the scaled canopy temperature, *T_a_* [°C] was the air temperature, both measured at time *t*, while *T_ll_* represented the lower limit temperature (temperature of a well-watered plant canopy), and *T_ul_* represented the upper limit temperature (temperature of a completely water-stressed plant canopy).

Pre-established _i_CWSI thresholds were entered into ARSPivot software at the beginning of the irrigation season and were based on historical data (O’Shaughnessy and Evett, 2010; O’Shaughnessy et al., 2015). Irrigations were triggered when the calculated _i_CWSI for a specific MZ (treatment plot designated with a “C”) exceeded one of three tiers (**Table 2**). The irrigation depth applied was dependent on the tier that included the calculated _i_CWSI.

**Table 2:** Tiers of integrated CWSI (_i_CWSI) thresholds and irrigation depths for cotton managed in 2021 and 2022 by the ISSCADA system in Bushland, Texas.

Crop water stress level	No Stress	TIER I- Minimum Stress	Tier II- Medium Stress	Tier III- Maximum Stress
_i_CWSI Threshold	_i_CWSI< 150	150 ≤ _i_CWSI<250	250 ≤ _i_CWSI< 325	_i_CWSI ≥ 325
Prescribed Irrigation Depth (mm)				
Irrigation Levels	Withhold Irrigation	Minimum	Medium	Maximum
75	0	17	25	35
50	0	12.5	17	25
25	0	7	12.5	17

##### 2.4.2.2 Hybrid

One soil water sensing station was deployed in each of three plots- 5, 8 and 19 in 2021 and plots 25, 37, and 19 in 2022, providing input for replications designated H_75_, H_50_, and H_25_, respectively. Irrigations were triggered for each treatment replicate using the combination of soil water depletion (SWD) and _i_CWSI thresholds for the designated treatment level. As an example, in 2021, irrigation scheduling for all plots designated H_75_ (plots 5, 14, 25 and 34) used input from the soil water sensor located in plot 5 and an _i_CWSI calculated over all four plots for decision support. Total SWD was calculated as the summation of soil water depleted from each layer of soil sensed by the four TDRs, equation 2:


(2) $$SWD=\;\sum_{i=1}^{4}l_{i}\left(\frac{\theta_{FCi}-\;\theta_{vi}}{\theta_{FCi}-\theta_{PWPi}}\right)$$

*l_i_* was the thickness of the soil layer *i*, θ*_FCi_* was field capacity of layer *i*, θ*_v_* was volumetric soil water content of layer *i*, and θ_PWPi_ was permanent wilting point for layer *l*. Since most of the water extracted by cotton is in the top 40 cm (Pabuayon et al., 2019), the TDRs were installed at depths of 10, 20, 30 and 50 cm for which field capacity was set to 0.35, 0.34, 0.33 and 0.33 m^3^ m^-3^, respectively. Permanent wilting point was set to 0.18 m^3^ m^-3^ for each layer based on information from Unger (1996); Schwartz et al. (2003); Heng et al. (2009), and Tolk and Evett (2012). If SWD ≤ 0.10 then irrigation was withheld; if SWD ≥ 0.65, fraction of depletion for cotton (Allen et al., 1998), then the maximum irrigation depth for the designated treatment level was applied (**Table 2**). However, if the 0.10< SWD< 0.65, then irrigation was applied according to the _i_CWSI thresholds for the designated treatment level (**Table 2**). If no soil water readings were available (e.g., sensor or communication failure), the ISSCADA system relied on the _i_CWSI thresholds for irrigation scheduling.

**Figure 3:**
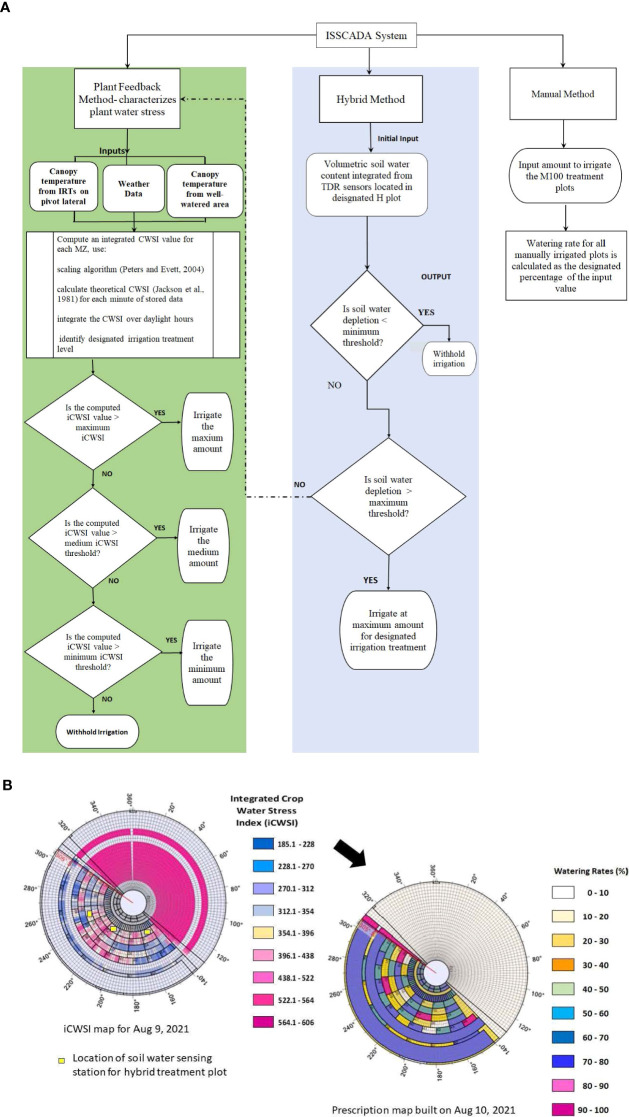
Summary of: **(A)** decision making algorithm used in the ISSCADA system, and **(B)**_i_CWSI map built from the Aug 9, 2021, scan showing qualitative information from soil water sensing stations, and prescription watering map built from data captured during the scan.

Variable rate irrigation was initiated on Jul 8, 2021, and on Jul 15, 2022, by running a scan (action whereby the sprinkler travels across the field without irrigating) using the ISSCADA system. The following day, the ISSCADA system built a prescription map. Thereafter, a scan was run every 3 to 4 days during a seven-day period. **Figure 3** summarizes the irrigation scheduling algorithms operated by the ISSCADA system and presents an example of an _i_CWSI map with qualitative SWD information from soil water sensing stations located in “H” designated plots- plot 5 (H_75_), plot 8 (H_50_), and plot 19 (H_25_). The yellow-colored square symbols indicated that the soil water depletion levels were between the minimum and maximum SWD thresholds. In this example, the prescription map was built automatically after midnight on Aug 10, 2021, after a scan was executed on Aug 9, 2021.

### 2.5 Calculations

Heat units (HU, °C) were calculated to characterize the growing environment for cotton using equation 3:


(3) $$HU=\;\frac{\left(T_{max}-\;T_{min}\right)}{2}-T_{b}$$

where *T_max_*, *T_min_* are the maximum and minimum daily air temperature, respectively and 15.6°C was used as the base temperature (*T_b_*), typical for the study region (Mahan et al., 2014; Masasi et al., 2020).

Seasonal evapotranspiration (ET_c_) was calculated for the growing season using the soil water balance equation 4:


(4) $$ET_{c}=I+P+F-\;\Delta S-R$$

where *I* was cumulative irrigation, *P* was seasonal precipitation, Δ*S* was the change in soil water content from the first to the last NP measurement to a depth of 2.3 m, F was flux across the lower boundary of the control volume, and R was runoff, all units were in mm. Values of F were assumed to be zero as NP measurements did not indicate a change in flux in soil water content below 170 cm (data not shown).

#### 2.5.1 Irrigation efficiency equations

Crop water productivity:


(5) $$CWP=(\frac{Lint\;and\;seed\;yield}{P+I+\Delta s})\;(kg\;m^{3})$$

Irrigation water use efficiency as described by Howell (2002):


(6) $$IWUE=\;\frac{Cotton\;seed\;yield_{p}-Cotton\;seed\;yield_{M0}}{Seasonal\;ETc_{p}-Seasonal\;ETc_{M0}}\;\left(kg\;m^{3}\right)$$

where *p* is any treatment plot other than those designated as M_0_.

#### 2.5.2 Statistical methods

The impact of deficit irrigation on cotton response was evaluated by ANOVA using PROC GLM (SAS version 9.4, Carry, NC). Significant differences between means of the deficit irrigation method by irrigation level treatments were compared individually with those of the fully irrigated treatments (M_100_) using Dunnett’s t Test (Carmer and Walker, 1985; Lee and Lee, 2018). Effects of main treatments (irrigation level and irrigation scheduling method) and their interaction on cumulative irrigation and crop response were analyzed using PROC GLM (SAS version 9.4, Carry, NC) with the least squares means method, and adjusted for multiple comparisons using the Tukey HSD test.

## 3 Results

### 3.1 Climate and postplant irrigations

Cumulative precipitation amounts from May through October of each year were 305 mm and 326 mm for the 2021 and 2022 growing seasons, respectively (**Table 3**). However, the pattern of rainfall was significantly different between the two years. Rainfall from January through May of 2021 totaled 145 mm. The total rainfall in May occurred prior to planting, thereafter, measurable rainfall occurred approximately every 14 days. Monthly cumulative precipitation for August and September were smaller than the average values for these months. In 2022, total precipitation from January through the first half of May was less than 32 mm. While precipitation in this second growing season was greater than 2021, approximately 42% occurred on July 29 and July 30. Outside of these two events, 86% of all rainfall events for the season were recorded to be less than 10 mm. Except for the month of July, monthly cumulative precipitation was smaller than the 58-year average precipitation amounts for the study location (O’Shaughnessy et al., 2014). Preplant precipitation in 2021 provided adequate moisture in the soil profile, avoiding excessive postplant irrigations. However, the relatively cool air temperatures in June and July slowed crop maturation. Even so, the overall limited precipitation during the growing season aided the ISSCADA system in detecting different levels of crop water stress. In 2022, the paucity of precipitation in the winter and spring required postplant irrigations to germinate and establish a uniform stand.

**Table 3:** Monthly precipitation and mean climatic values for the 2021 and 2022 growing seasons in Bushland, Texas.

Growing Season	Precipitation (mm)	Min Air Temp (°C)	Max Air Temp (°C)	Min RH (%)	Max RH (%)	Wind Speed (m s^-1^)
2021						
May	87	11.3	24.1	40.3	91.8	4.9
June	40	17.2	31.6	30.8	84.3	3.8
July	107	18.1	31.0	38.2	90.5	3.3
August	16	18.2	31.6	33.5	85.9	4.0
September	26	14.7	29.9	29.3	80.2	4.4
October	29	7.8	23.7	25.5	77.0	4.2
2022						
May	23	11.3	29.7	17.3	69.1	5.7
June	20	17.7	32.3	28.6	74.5	5.2
July	159	20.2	35.2	25.1	72.5	3.8
August	59	18.5	30.4	38.4	84.6	3.2
September	3	14.9	30.3	29.0	80.5	4.2
October	48	8.0	20.9	36.5	84.2	3.7

Postplant irrigations were applied to establish a uniform stand; these irrigations totaled 38 mm and 95 mm in 2021 and 2022, respectively (**Table 4**). The uniform postplant irrigations were greater in 2022 for two reasons- drought conditions prevailed in the winter and spring months of 2022, and secondly, the VFD for the center pivot pump failed in early June (after planting), and two uniform irrigations were applied prior to replacing the equipment to assure adequate soil moisture during the down time. Irrigations were terminated in the treatment plots designated M_0_, when VRI applications were initiated on July 10, 2021, and July 15, 2022. Soil water sensing stations were deployed on July 7, 2021, and July 12, 2022, in the appropriate ISSCADA-hybrid treatment plots.

**Table 4:** Comparison of mean cumulative irrigation, seed cotton yield, and IWUE of each deficit irrigated treatment with the M_100_ and M_0_ control treatments using Dunnett’s t test to indicate significant differences between means at the p ≤ 0.05.

Year: 2021	Cumulative Irrigation (mm)		Seed Cotton Yield (kg ha^-1^)		IWUE (kg m^-3^)	
Control M_0_	38 ± 0		1069 ± 342		–	
Control M_100_	356 ± 0		2069 ± 729		0.31 ± 0.20	
Deficit Treatment	Difference in means^1^	p values	Difference in means^2^	p value	Difference in means^3^	p value
M_75_	-85	<0.0001^*^	+566	0.423	+0.36	0.5074
M_50_	-168	<0.0001^*^	-19	1.000	+0.32	0.6482
M_25_	-243	<0.0001^*^	-544	0.467	+0.36	0.5337
C_75_	-138	<0.0001^*^	+274	0.960	+0.42	0.3583
C_50_	-180	<0.0001^*^	-147	0.999	+0.33	0.6157
C_25_	-226	<0.0001^*^	-476	0.611	+0.30	0.6989
H_75_	-77	<0.0001^*^	+102	1.000	+0.15	0.9915
H_50_	-169	<0.0001^*^	+51	1.000	+0.40	0.4123
H_25_	-217	<0.0001^*^	-458	0.650	+0.22	0.9089
Year 2022	Cumulative Irrigation (mm)		Seed Cotton Yield (kg ha^-1^)		IWUE (kg m^-3^)		
Control M_0_	95 ± 0		1960 ± 490		–	
Control M_100_	324 ± 0		2187 ± 255		0.12 ± 0.11	
M_75_	-52	<0.0001^*^	+360	0.8220	+0.29	0.9894
M_50_	-100	<0.0001^*^	+208	0.9901	+0.42	0.9799
M_25_	-157	<0.0001^*^	-460	0.6054	-0.49	0.6702
C_75_	-58	<0.0001^*^	+130	0.9997	+0.26	0.9999
C_50_	-95	<0.0001^*^	-115	0.9999	+0.13	1.0000
C_25_	-134	<0.0001^*^	-320	0.8929	-0.07	0.9998
H_75_	-65	<0.0001^*^	+240	0.9762	+0.34	0.9972
H_50_	-133	<0.0001^*^	-221	0.9860	+0.01	1.000
H_25_	-155	<0.0001^*^	-320	1.000	-0.20	0.9857

Difference in means = (M, C or H)_x_ - M_100_, where x = 75, 50, 25, and M, C, and H are the irrigation scheduling methods- manual, ISSCADA-plant feedback and ISSCADA-hybrid, respectively.

^1^A negative value indicates the mean amount of water saved; a positive value indicates irrigation in excess of the control treatment mean M_100_.

^2^A positive value indicates yields greater than, and a negative value indicates yields less than that for the control treatment M_100_.

^3^A positive value indicates that IWUE was improved over the control treatment M_100_, and a negative value indicates that mean seed cotton yield was less than that of the M_0_ treatment

^*^Indicates a significant difference between the means at p ≤ 0.05.

**Figure 4:**
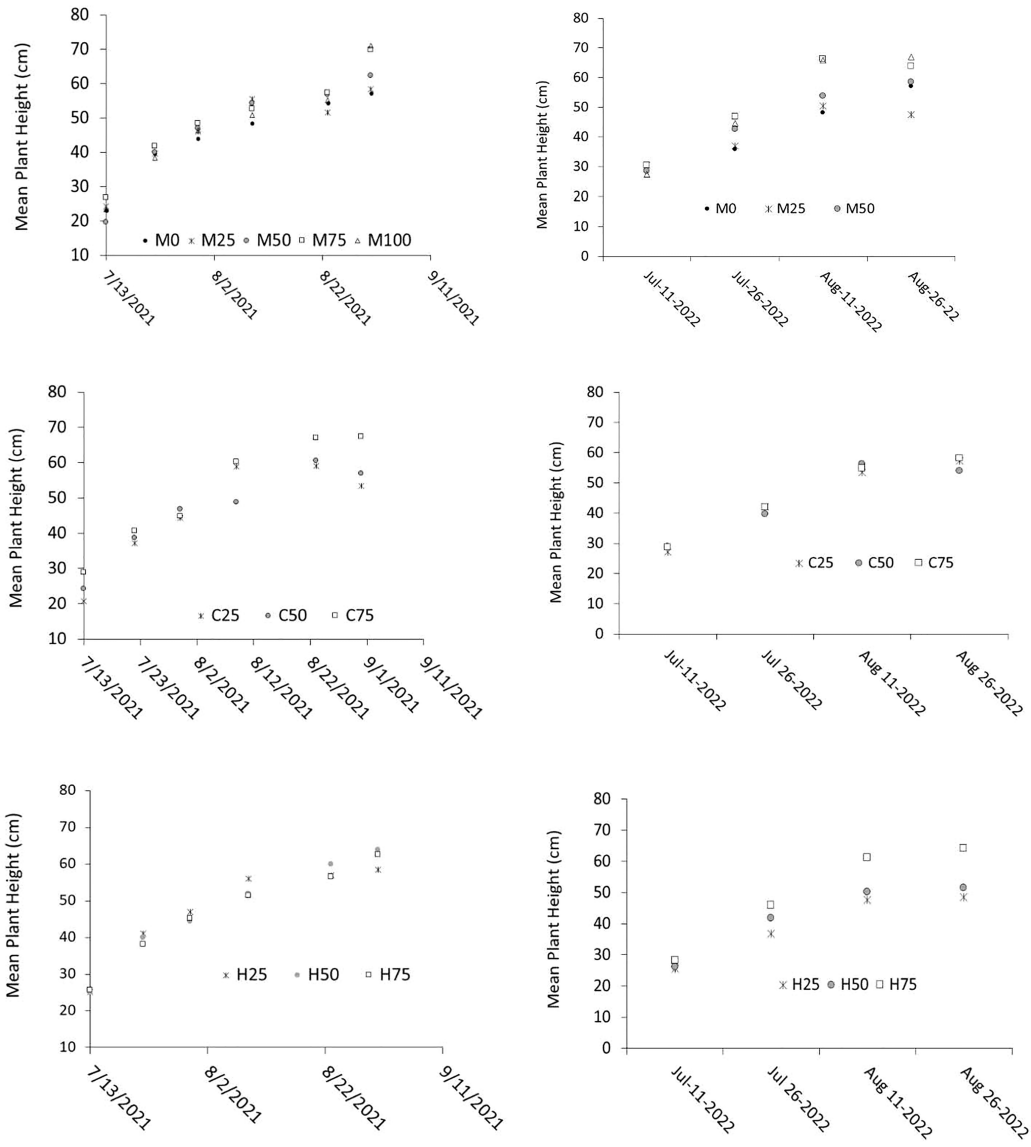
Mean plant heights and growth stage observations for the 2021 growing season (left); and the 2022 growing season (right), for the manual (M), ISSCADA-plant feedback (C), and the ISSCADA-hybrid (H) method.

Plant height is a good indicator for vegetative cotton growth (Ritchie et al., 2010; Zhou and Yin, 2014). Throughout the growing season, mean plant heights in 2021 were closely clustered among irrigation levels for the manual and ISSCADA-hybrid irrigation scheduling treatment plots until the end of the irrigation season. The increase in plant heights at the end of the season could have resulted from the last two irrigations at the end of the season (**Figure SM-1**). Plant heights in the ISSCADA-plant feedback method showed distinct heights among irrigation levels beginning Aug 12 (**Figure 4**). In 2022, plant height appeared to increase quickly after the intensive rainfall event of July 30, indicating luxury vegetative growth between Jul 26 and Aug 11 (**Figure 4**). Apart from the H_75_ treatment plots, scans executed on Aug 3 and Aug 6 resulted in withholding irrigations on Aug 4 and Aug 7 in all ISSCADA-plant feedback treatment plots and in the ISSCADA-hybrid treatment plots at the I_50_ and I_25_ levels (**Figure 1**. SM- 2). The irrigation amount prescribed for the H_75_ treatment plots resulted from the _i_CWSI threshold value as the calculated SWD level was between the minimum and maximum thresholds. Irrigations were terminated on Sep 9, 2021, and on Aug 26, 2022, after observing that most plants had at least five vegetative nodes above first white flower. Although a scan was executed on Aug 11, 2022, irrigations were only recommended for the H_75_ treatment plot. The last irrigation applied on Aug 26 was erroneously applied as a uniform irrigation of 11.4 mm.

### 3.2 Deficit irrigated crop response compared with fully and extreme deficit treatment plots

The M_100_ and M_0_ treatment plots were established as control treatments for determining maximum irrigation amounts and evaluating IWUE, respectively. Individual comparisons of mean cumulative irrigation and mean crop response of seed cotton yield and IWUE were made between the M_100_ control treatment (**Table 4**) and means from each type of deficit irrigation level X irrigation scheduling treatment. Due to differences in climatic conditions, data were analyzed separately by year. Of interest when comparing mean differences were reductions and overages in irrigation applied, gains and losses in seed cotton yield, and improvements in IWUE. In 2021, the comparisons showed that mean cumulative irrigations in the deficit irrigated treatments were significantly less as compared with the M_100_ treatment, yet mean seed cotton yields were not significantly different at p< 0.05. This indicated that no significant yield loss had occurred between any of the deficit irrigation treatments and the fully irrigated treatment. Mean IWUE values were numerically greater in all the deficit irrigated treatments, but not significantly different from the M_100_ treatment (at p< 0.05). In 2022, mean cumulative irrigations for the deficit irrigated treatment plots were again significantly less than the mean for the M_100_ treatment. Mean seed cotton yields were statistically similar as compared with the M_100_ treatment, however, mean yields were not sustained in the M_25_, C_50_, C_25_, H_50_ and H_25_ treatments. Mean IWUE values were not significantly different from the M_100_ treatment, though IWUE means were numerically greater in all I_75_ irrigation level by irrigation scheduling treatments. The mean seed cotton yield in the M_0_ treatment was only 11% less than that in the M_100_ treatment and was relatively large, due to the seed cotton yield measured in plot 1, which was at the 90^th^ quantile of the distribution of harvested values within the M_0_ treatment. Removing this value as an outlier, increased mean IWUE values for all treatments, although the adjusted differences were not significantly different compared with the M_100_ treatment. Means in **Tables 4**–**6** include all measured values.

### 3.3 Crop response compared between irrigation level and method

Pairwise means were compared to investigate differences between irrigation level, irrigation method and their interaction at the I_75_, I_50_ and I_25_ levels. Data were analyzed separately for each growing season. In 2021, when data were grouped by irrigation level, cumulative irrigation amounts and ET_c_ were significantly different across the three irrigation levels (**Table 5**). Overall seed cotton yield was significantly less in the I_25_ treatment level. Mean IWUE was not significantly different among irrigation levels. Grouping the data by irrigation scheduling method demonstrated that scheduling methods had a significant impact on cumulative irrigation amount and ET_c_ in the ISSCADA-plant feedback treatment plots, resulting in significantly less water applied to and significantly smaller ET_c_ values in these plots (**Table 5**). However, mean seed cotton yield, CWP and IWUE were statistically identical among scheduling methods.

**Table 5:** Mean and standard deviation values for cumulative irrigation and crop response parameters by irrigation level and irrigation scheduling method for cotton grown at Bushland, Texas in the 2021 growing season.

Irrigation Treatment	Irrigation Scheduling Method	Cumulative Irrigation (mm)	Seed Cotton Yield (kg ha^-1^)	ET_c_ (mm)	CWP (kg m^-3^)	IWUE (kg m^-3^)
Averages Across Irrigation Level								
I_75_	–	256 ± 32 a*	2383 ± 452 a	570 ± 45 a	0.42 ± 0.08	0.62 ± 0.24
I_50_	–	184 ± 18 b	1960 ± 379 a	492 ± 27 b	0.41± 0.07	0.66 ± 0.23
I_25_	–	127 ± 16 c	1577 ± 366 b	438 ± 20 c	0.36 ± 0.08	0.60 ± 0.43
Averages Across Irrigation Scheduling Method								
	Manual	191 ± 69 ab	2070 ± 669	509 ± 77 ab	0.40 ± 0.09	0.66 ± 1.06
	ISSCADA-C	175 ± 43 b	1953 ± 445	480 ± 40 b	0.41 ± 0.08	0.66 ± 0.33
	ISSCADA-H	202 ± 61 a	1968 ± 429	511 ± 67 a	0.38 ± 0.07	0.57 ± 0.28
Averages Across Irrigation Level X Irrigation Scheduling Method								
(I_75_)	Manual	271 ± 0 a	2635 ± 588 a	597 ± 20a	0.44 ± 0.09	0.67 ± 0.26
	ISSCADA-C	218 ± 25 b	2343 ± 345 ab	520 ± 23b	0.45 ± 0.08	0.73 ± 0.24
	ISSCADA-H	279 ± 0 a	2171 ± 373 ab	592 ± 38a	0.37 ± 0.05	0.46 ± 0.15
(I_50_)	Manual	188 ± 0 bc	2050 ± 636 ab	505 ± 35bc	0.40 ± 0.10	0.63 ± 0.36
	ISSCADA-C	176 ± 29 cd	1922 ± 118 ab	477 ± 29bcde	0.40 ± 0.04	0.64 ± 0.16
	ISSCADA-H	187 ± 0 bc	2120 ± 286 ab	495 ± 7 bcd	0.43 ± 0.05	0.71 ± 0.19
(I_25_)	Manual	113 ± 14^1^ e	1525 ± 265 b	424 ± 16e	0.36 ± 0.07	0.67 ± 0.42
	ISSCADA-C	130 ± 16 e	1593 ± 466 b	443 ± 17de	0.36 ± 0.10	0.61 ± 0.55
	ISSCADA-H	139 ± 0 de	1611 ± 446 b	447 ± 21cde	0.36 ± 0.09	0.53 ± 0.43

*Groups of means followed by a different letter are significantly different at p ≤ 0.05.

^1^A logistical error prevented an irrigation in plot 6 early in the season.

Analyzing all sets of pairwise means for irrigation level by irrigation scheduling method indicated that mean cumulative irrigation for the C_75_ treatment plots was significantly less compared with the M_75_ and H_75_ treatments. At the I_50_ and I_25_ levels, mean cumulative irrigations were similar among irrigation scheduling methods. Mean differences in ET_c_ response followed the same statistical trend as differences in cumulative irrigation. Mean seed cotton yields were only significantly greater between the M_75_ treatment and all irrigation scheduling methods at the I_25_ level. Mean values of CWP and IWUE were not statistically different among all irrigation level X irrigation scheduling treatments.

In 2022, as in the previous season, results showed that when data were grouped by irrigation level, overall mean cumulative irrigation and ET_c_ values were significantly different. However, overall mean seed cotton yield, CWP and IWUE were not affected by irrigation level (**Table 6**). Aggregating data by irrigation scheduling method showed that mean cumulative irrigations applied to the ISSCADA-hybrid treatment plots were less compared with the manual and ISSCADA-plant feedback treatment plots. However, mean seed cotton yield, ET_c_, CWP and IWUE were statistically identical among irrigation scheduling methods.

**Table 6:** Mean and standard deviation values for cumulative irrigation and crop response parameters by irrigation level and method and for cotton grown at Bushland, Texas in the 2022 growing season.

Irrigation Treatment	Irrigation Scheduling Method	Cumulative Irrigation (mm)	Seed Cotton Yield (kg ha^-1^)	ET_c_ (mm)	CWP (kg m^-3^)	IWUE (kg m^-3^)
Averages Across Irrigation Level						
I_75_	–	266 ± 10 a*	2430 ± 460	631 ± 45 a	0.38 ± 0.08	0.28 ± 0.28
I_50_	–	215 ± 22 b	2145 ± 403	572 ± 27 b	0.38± 0.07	0.15 ± 0.38
I_25_	–	175 ± 13 c	1819 ± 440	531 ± 20 c	0.33 ± 0.08	-0.17 ± 0.57
Averages Across Irrigation Scheduling Method						
	Manual	223 ± 46 a	2223 ± 483	586 ± 77	0.39 ± 0.09	0.12 ± 0.40
	ISSCADA-C	227 ± 36 a	2085 ± 412	580 ± 40	0.35 ± 0.08	0.85 ± 0.36
	ISSCADA-H	206 ± 40 b	2085 ± 596	566 ± 67	0.36 ± 0.07	0.05 ± 0.60
Averages Across Irrigation Level X Irrigation Scheduling Method						
I_75_	Manual	272 ± 0 a	2547 ± 469	655 ± 49a	0.39 ± 0.06	0.33 ± 0.17
	ISSCADA-C	266 ± 16 a	2317 ± 396	620 ± 19ab	0.37 ± 0.06a	0.22 ± 0.25
	ISSCADA-H	259 ± 0 a	2427 ± 604	616 ± 37ab	0.39 ± 0.09	0.28 ± 0.37
I_50_	Manual	224 ± 9 b	2382 ± 304	574 ± 32abc	0.41 ± 0.03	0.34 ± 0.25
	ISSCADA-C	229 ± 22 b	2086 ± 289	570 ± 15bc	0.37 ± 0.05	0.10 ± 0.52
	ISSCADA-H	191 ± 0 c	1966 ± 546	560 ± 60bc	0.35 ± 0.08	0.01 ± 0.58
I_25_	Manual	167 ± 0 c	1727 ± 206	524 ± 26c	0.35 ± 0.07	-0.32 ± 0.28
	ISSCADA-C	190 ± 15 c	1867 ± 503	555 ± 89abc	0.31 ± 0.06	-0.07 ± 0.55
	ISSCADA-H	169 ± 0 c	1862 ± 630	522 ± 44c	0.35 ± 0.09	-0.13 ± 0.86

*Groups of means followed by a different letter are significantly different at p ≤ 0.05.

Analyses of the pairwise means for irrigation level by irrigation method showed that the mean cumulative irrigations at the I_75_ and the I_25_ irrigation levels were similar among irrigation scheduling methods. In the I_50_ treatment, cumulative irrigation for the ISSCADA-hybrid irrigation scheduling method was significantly smaller as compared with the manual and ISSCADA-plant feedback methods. Mean seed cotton yield, CWP and IWUE were similar for all irrigation level by irrigation scheduling treatments. Results were mixed for differences among mean ET_c_ values, where ET_c_ values for the M_25_ and H_25_ treatments were significantly less than all irrigation scheduling methods at the I_75_ level (**Table 6**).

## 4 Discussion

The performance of a GIS-based ISSCADA system was investigated to determine its feasibility as an automated deficit irrigation scheduling tool for cotton grown in a semi-arid region. Crop response- mean seed cotton yield, ET_c_, CWP and IWUE resulting from the two ISSCADA irrigation scheduling methods were compared with those from fully irrigated (M_100_) treatment plots. Performance of the two different ISSCADA irrigation scheduling methods (the plant feedback and hybrid) at irrigation levels of I_75_, I_50_ and I_25_ of full were also compared with a manual irrigation scheduling method using weekly NP readings. The latter comparison was made to investigate whether the ISSCADA system could perform similar or better than the accurate manual method of irrigation scheduling with a neutron probe (Gear et al., 1977), and to evaluate any advantage in using soil water sensing with the ISSCADA system.

In 2021, climatic evaporative demand in May and June were relatively low due to lower maximum daily air temperatures throughout May and rainfall in both months. Although only 4% less precipitation occurred in the 2021 growing season compared with the 2022 growing season, the patterns of rainfall were distinctly different. The large postplant irrigations and the intensive rainfall event in the 2022 growing season reduced IWUE and obscured the effects of irrigation treatments as compared with values from the 2021 growing season. However, since 67% of variable rate applications occurred prior to the intensive rainfall event and because physiological differences in plant growth were measurable just prior to this extreme event, it was valid to compare performance of the irrigation scheduling methods in both seasons. Moreover, in both years, it should be noted that cumulative irrigations were not significantly greater for the ISSCADA scheduling methods as compared with the manual method at any irrigation level. The analysis from the two-year study demonstrated that in both years, deficit irrigation at the I_75_ level by the manual (M) and by both ISSCADA irrigation-scheduling methods-maintained seed cotton yields that were similar compared with that of the fully irrigated treatment plots, while achieving significant water savings. Mean cumulative irrigations applied in 2021 were 24%, 39% and 22% less in the M_75_, C_75_ and H_75_ treatments, respectively, compared with the M_100_ treatment. At the I_50_ level, mean cumulative irrigations were 48%, 51% and 47% less compared with the M_100_ treatment. However, mean seed cotton yields for the M and C irrigation scheduling methods were less than the M_100_ treatment, indicating that at this lesser irrigation level, seed cotton yields could be penalized. Similarly, in 2022, the reduction in cumulative irrigation compared with the M_100_ treatment was 16%, 18% and 20% for the M_75_, C_75_, and H_75_ treatments, respectively. The reduction in seed cotton yield in the M_100_ treatment was not unexpected as some studies have shown that full irrigation or large amounts of untimely precipitation result in a decline of seed cotton yield (Wanjura et al., 2002; O’Shaughnessy and Evett, 2010; Sampathkumar et al., 2013).

Seed cotton yields at the fully irrigated and deficit irrigated levels from this study were consistent with yields from those reported by Bordovsky and Porter (2008) in Halfway, Texas, and at the CPRL by Colaizzi et al. (2009a) and O’Shaughnessy et al. (2015) after adjusting yields for seed weight. In this study, results at each deficit irrigation level for this study demonstrated that the ISSCADA irrigation methods performed in a manner similar with the manual irrigation scheduling method. This result was positive since the ISSCADA methods provided automated decision support, while the manual method was time consuming, required labor intensive use of an expensive and highly regulated neutron probe, and calculations to replenish crop water use to an established setpoint. The ISSCADA-plant feedback and ISSCADA-hybrid methods also performed in a similar manner to one another. This suggested that the ISSCADA-plant feedback method alone could be used to manage deficit irrigation scheduling at mild, moderate, and extreme deficit irrigation levels for cotton production in a semi-arid environment. While using the ISSCADA system unaccompanied by soil water sensors would save the cost and effort of installing soil water sensing instrumentation, it is, however, it is beneficial to include a second method of sensor feedback for redundancy. Soil water sensors can indicate the need for irrigation during periods of extended cloud cover when the ISSCADA-plant feedback method will not provide a trigger. Use of the TDRs with the ISSCADA system could replace NP readings for the purpose of practicing deficit irrigation management.

It is possible that the ISSCADA irrigation scheduling methods could be improved to increase CWP or IWUE by managing deficit irrigation according to crop growth stage (Bordovsky et al., 2015). Future studies could include the use of different types of plant sensors, such as spectral radiometers or imaging cameras, to help determine crop coefficients or crop growth stages to refine irrigation applications (Hunsaker et al., 2003; Dreccer et al., 2019). Modification of the ISSCADA algorithms to adjust irrigation timing and amounts based on the additional data streams would also be necessary, as well as field studies to test the feasibility of a modified system.

## Data availability statement

The raw data supporting the conclusions of this article will be made available by the authors, without undue reservation.

## Author contributions

SO’S and CB contributed to the conception and design of the study. SO’S performed the statistical analysis and wrote the first draft of the manuscript. PC reviewed the soil water sensing data and contributed to calculation and interpretation of soil water data. All authors contributed to the article and approved the submitted version.
