# Supplementary material for: Sensor feedback system enables automated deficit irrigation scheduling for cotton
Source: Front Plant Sci. 2023 Mar 9;14:1149424. doi: 10.3389/fpls.2023.1149424 (PMC10034034; doi:10.3389/fpls.2023.1149424)
Supplement: Supplementary file 1 [file DataSheet_1.docx]

**Supplementary Material**

Volumetric soil water content from the Acclima TDRs in the top 500 mm of the soil profile was converted to soil water depletion. Daily average soil water depletion values are shown in Figure SD-1 for 2021 and SD-2 for 2022. Missing data and readings reported as NAN (not a number) for time intervals of 15 min to 2 hours were estimated using linear interpolation. If missing data intervals were greater, NP readings and daily ET estimates from ET_o_ X K_c_ values were used to fill daily data gaps.

The near instantaneous reduction in soil water depletion in plot 25 (H_75_ treatment) (SD-2), occurred after a rainfall event totaling 20.8 mm. However, the response was unlike that in plot 37 (H50 treatment) and in plot 19 (H_25_ plot). All four TDRS in plot 25 recorded a change in percent volumetric water content, ranging from 22% in the TDRs installed in the upper soil layers, while the percent change in the two deepest TDRs was 15.6% and 10.4% at the 30 cm and 50 cm depths, respectively. It is probable that cracks in the soil near the probes led to preferential flow.

|  |
| --- |
| Figure SM-1. Soil water depletion for the 2021 growing season from TDRs installed in the ISSCADA-hybrid irrigation scheduling treatment plots. |

|  |
| --- |
| Figure SM- 2. Soil water depletion as determined from the TDR readings for the 2022 growing season from ISSCADA-hybrid (H) treatment plots. |
